# Environmental Antimicrobial Resistance: Key Drivers, Hotspots, Innovative Strategies, and Challenges in the Fight Against Superbugs

**DOI:** 10.1002/mbo3.70067

**Published:** 2025-10-14

**Authors:** Kindu Alem, Mulat Dagnew, Mucheye Gizachew, Baye Gelaw, Feleke Moges

**Affiliations:** ^1^ Department of Medical Microbiology School of Biomedical and Laboratory Sciences, College of Medicine and Health Sciences University of Gondar Gondar Ethiopia; ^2^ Biology Department Faculty of Natural and Computational Sciences Woldia University Woldia Ethiopia

**Keywords:** antibiotic resistance genes, antimicrobial resistance, environmental hotspots, superbugs

## Abstract

Currently, one of the major global public health concerns that impacts humans, animals, and the environment is antimicrobial resistance (AMR). It poses serious problems for contemporary medicine by casting doubt on the effectiveness of antibiotics and other antimicrobials. The main causes of environmental AMR include the abuse of antibiotics and nonantibiotic substances, such as biocides and heavy metals, in veterinary care, agriculture, and healthcare, which hasten the generation of resistant superbugs. The dissemination of drug‐resistant microbes and genetic elements is aided by environmental hotspots, such as hospitals, wastewater treatment facilities, livestock farms, and pharmaceutical production facilities. The Global Action Plan, National Action Plan, antimicrobial stewardship initiatives, improved surveillance, precise diagnostics, infection prevention and control, and the concept of One Health are all components of the multidimensional strategy needed to combat AMR. Promising alternative therapies to conventional treatments include antimicrobial peptides, bacteriophage therapy, immunotherapies, nanotechnology, probiotics, clustered regularly interspaced short palindromic repeats‐Cas system, and artificial intelligence. The concept of One Health, recognizing the interdependence of human, animal, and ecosystem health, offers a holistic strategy for addressing AMR. However, implementing these strategies presents significant challenges, including resource limitations and regulatory barriers. This review tries to give a complete picture of AMR by focusing on the main factors that cause it and the hotspots that promote the acquisition of resistance genes. Additionally, it examines approaches to counteract AMR and the obstacles encountered in their execution.

## Graphical abstract

Antimicrobial resistance (AMR) poses a critical threat to global health, affecting humans, animals, and the environment. This review explores major drivers and hotspots of AMR and outlines innovative strategies, including One Health–based interventions, to mitigate the spread of resistant pathogens and address this escalating worldwide health challenge.

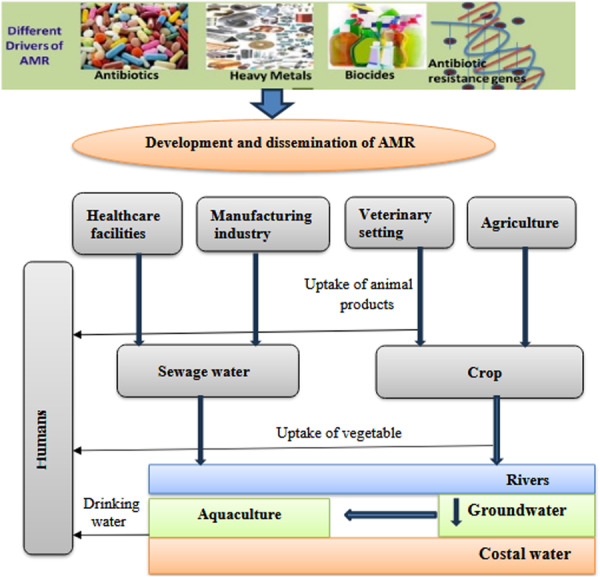

## 1 Introduction

Antimicrobial resistance (AMR) has emerged as a significant global issue in the 21st century, endangering the environment, animals, and humans in interdependent ecosystems. Superbugs, bacteria that have developed resistance to multiple antibiotics, such as multidrug‐resistant (MDR) *Mycobacterium tuberculosis* (*M. tuberculosis*), carbapenem‐resistant Enterobacteriaceae, and methicillin‐resistant *Staphylococcus aureus* (*S. aureus*), pose a serious threat to ecological stability, food security, and contemporary medicine because of their rapid emergence and spread (Ferraz 2024; Tang et al. 2023). AMR arises when bacteria acquire resistance to antimicrobial agents as a result of genetic changes or lateral gene transfer, such as transformation, transduction, and conjugation (Christaki et al. 2020).

Numerous channels, including human and animal waste, agricultural runoff, industrial effluents, and contaminated water systems, contribute to the spread of resistant bacteria and their genes. Therefore, antibiotic‐resistant bacteria (ARB) and genes linked to antibiotic resistance are both stored in and transmitted through the environment (Ajayi et al. 2024). Though, still, it is unknown how far AMR has evolved, how it spreads, and how persistent it is across different environmental settings. It has become increasingly evident that ARB and antibiotic‐resistant genes (ARGs) are dispersed throughout the environment, humans, and animals. The AMR dilemma is made worse by the constant spread of ARB and their resistance genes among the three domains: the environment, humans, and animals, which calls for prompt, concerted global action (Mudenda et al. 2023).

AMR has significant health effects, including increased morbidity and mortality, treatment failures, and raising healthcare costs. Treatment for a common infection caused by resistant bacteria, such as bloodstream, pneumonia, and urinary tract infections, has grown more challenging, and patients with impaired immune systems are at particular risk (Alara and Alara 2024). A study has revealed that bacteria like *Salmonella*, *Escherichia coli* (*E. coli*), and *Klebsiella pneumoniae* (*K. pneumoniae*) can reduce the efficacy of infection therapy in clinical, veterinary, and environmental settings (Aijaz et al. 2023). In addition to endangering human health, the environmental development of antibiotic resistance is also threatening food safety, fish farming, and livestock productivity. Both natural and human‐caused factors have contributed significantly to this rise, with the overuse and inappropriate handling of antibiotics in fish farming, agriculture, and healthcare subjecting resistant strains to selective pressure (Reghukumar 2023). Nonantibiotic antimicrobial agents like metals, biocides, herbicides, and pesticides also contribute to the dissemination of ARGs among bacterial species, including clinically significant bacteria (S. S. Chung et al. 2018).

AMR hotspots are areas where resistant microorganisms and ARGs are highly concentrated and actively transmitted (Ding et al. 2023). Major hotspots include hospitals and healthcare facilities, where high antimicrobial use (AMU) and effluent discharge contribute to resistance accumulation; pharmaceutical manufacturing sites, which discharge elevated levels of antimicrobials into water bodies; wastewater treatment plants, which serve as reservoirs and dissemination points for resistant bacteria; and urban and natural water systems, including rivers, lakes, and oceans, where resistant bacteria and ARGs circulate and spread to other ecosystems, are the major hotspots for AMR. These hotspots facilitate the horizontal exchange of resistance genes among microbial communities, amplifying AMR transmission at the human–animal–environmental interfaces (Abosse et al. 2024).

Integrated, cross‐sectoral approaches that take into account the environment, animal health, and human health are needed to combat AMR. To reduce AMR, worldwide solutions have been put forth, such as the Global Action Plan and the National Action Plan, antimicrobial surveillance (AS) and stewardship programs, effective diagnosis, the One Health concept, and innovative alternative therapies (Sangeda et al. 2020). However, several major obstacles impede its implementation, such as the burden of disease, limited laboratory capacity, inadequate funds, lack of behavior change, and inadequate government commitment. The interdependence of human, animal, and environmental health necessitates interdisciplinary collaboration to develop and execute effective AMR control strategies (Al‐Worafi 2024). Addressing the crisis requires identifying transmission pathways, understanding key drivers, and overcoming barriers in the environmental development and spreading of AMR (Endale et al. 2023). This review aims to explore a crucial dimension of AMR by examining, including its major drivers, environmental hotspots that facilitate resistance gene acquisition, and effective strategies for combating its spread. Additionally, it addresses the challenges associated with implementing these strategies in diverse environmental settings.

## 2 Evolution and Dissemination of AMR in the Environment

The World Health Organization (WHO) has listed AMR as one of the top 10 public health hazards and a fast‐expanding global issue. When bacteria evolve defenses against antimicrobial therapy, AMR develops, making these medications ineffective. A critical aspect of AMR is where bacterial pathogens evolve resistance to antibiotic drugs, which presents major difficulties for medical treatment (Estany‐Gestal et al. 2024). MDR bacteria, capable of resisting multiple classes of antibiotics, are particularly concerning, as some strains demonstrate resistance to almost all existing antibiotics. This resistance arises through intrinsic, acquired, and adaptive mechanisms, which allow bacteria to survive antimicrobial selection pressures (Lee et al. 2019).

**Figure 1:**
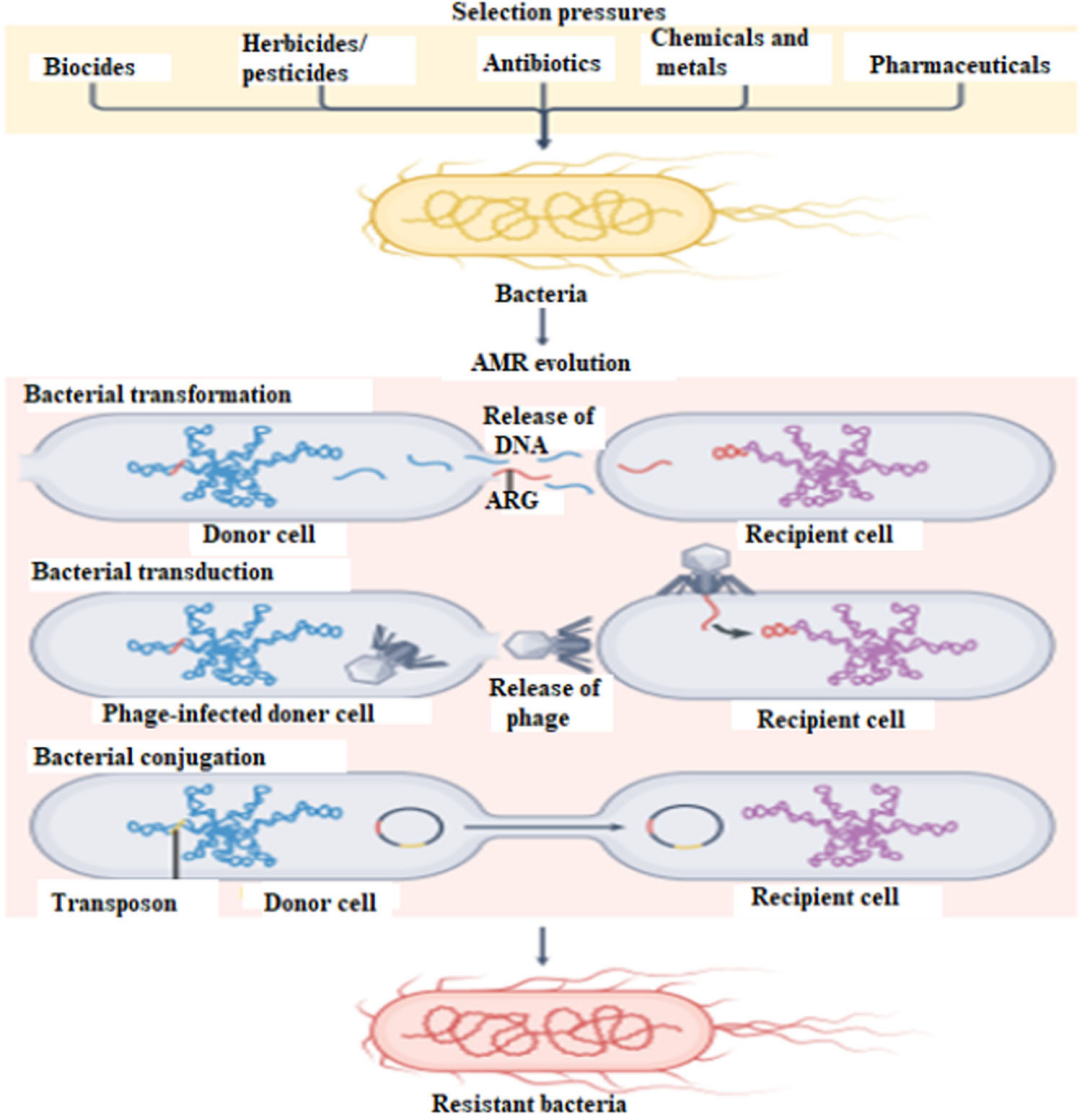
The emergence and spread of antibiotic resistance. Through the three main processes of transformation, transduction, and conjugation, antimicrobial selection pressures, such as antibiotics, metals, biocides, pesticides, and chemicals drive genetic adaptation in bacteria. Antimicrobial resistance (AMR) in bacterial populations is easier to evolve and spread because of these processes, which allow bacteria to acquire and transfer genetic material, including antibioticresistant genes (ARGs). Moreover, transposons, gene cassettes, and insertion sequences can mobilize pre‐existing resistance genes from a variety of bacterial flora and integrate them into extra genetic materials, such as plasmids. As a result, they are more able to move between bacterial cells, which speed up the spread of AMR (Djordjevic et al. 2024).

Intrinsic resistance is the innate ability of bacteria to resist particular antibiotics, which is caused by chromosomal genes that produce multidrug efflux pumps. This type of resistance is not acquired through mutations or transfer of external genetic material but rather exists as a part of the bacterium's normal physiology (Urban‐Chmiel et al. 2022). In contrast, acquired resistance occurs when bacteria gain resistance traits through genetic mutations or horizontal gene transfer (HGT), including mechanisms like transformation (taking up foreign genetic material from the external environment and integrating it into its genetic blueprint), transduction (transfer of genetic material by phages), and conjugation (direct exchange of hereditary material between two adjacent bacterial cells). The dissemination of ARGs among bacterial populations is largely driven by lateral gene transfer (Figure 1) (Djordjevic et al. 2024). Conversely, adaptive resistance arises temporarily in reaction to environmental stressors, such as subinhibitory antibiotic concentrations, nutrient deficiencies, or changes in pH and ion concentrations. Unlike intrinsic and acquired resistance, adaptive resistance returns to its initial state when the selective pressure is removed (da Cruz Nizer et al. 2021).

An important reservoir and route for the development and spread of AMR is the environment. Natural ecosystems such as water, soil, crops, and air harbor diverse bacterial communities, including those able to cause infection in humans and animals. Human actions greatly contribute to the widespread dissemination of AMR in the environment (Sharma et al. 2024). Pharmaceutical residues, hospital and industrial waste, as well as agricultural practices involving intensive crop and livestock farming, introduce antibiotics and ARGs into environmental matrices. These pollutants, including antibiotic residues, antibiotic‐resistant microbes, and drug resistance genes, often enter ecosystems through sewage and wastewater effluents, creating hotspots for AMR development. The regular movement of resistant bacteria and their genetic material between human, animal, and environmental systems increases the likelihood that resistance will evolve and spread (Graham et al. 2019). Mobile genetic elements (MGEs) like plasmids and transposons allow lateral gene transfer, which is the main way that genes causing antibiotic resistance spread. Antimicrobial‐resistant genes can be transferred between pathogenic and nonpathogenic bacteria in biological and environmental systems through conjugation, a crucial HGT mechanism. The development of resistance characteristics is further encouraged by the selective pressures exerted by metals, biocides, and antibiotic residues in the environment, which enable bacteria to endure in the presence of antibiotics and other stressors (C. Liu et al. 2022).

## 3 Drivers of Antimicrobial Resistance

**Figure 2:**
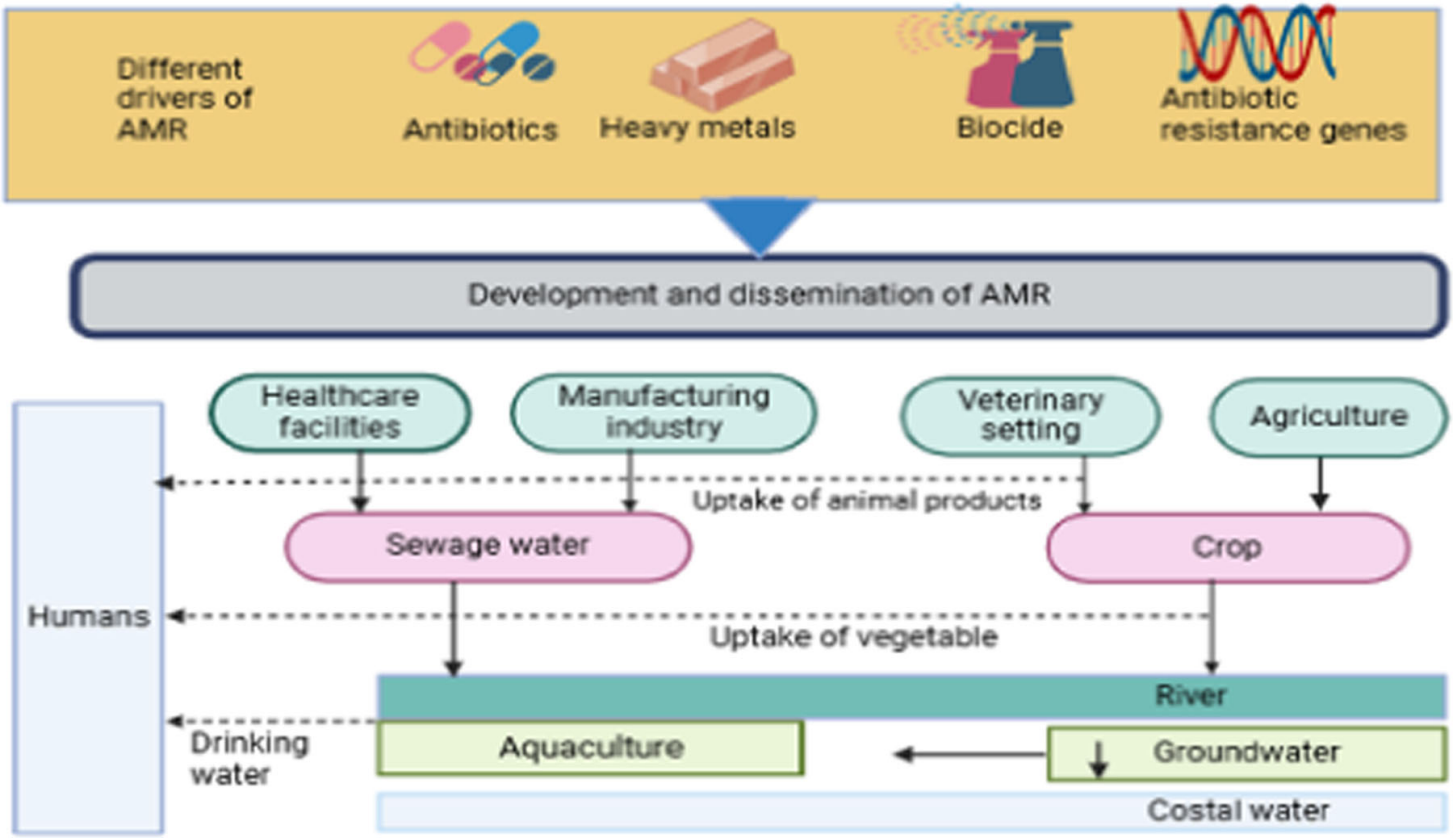
Potential pathways of antibiotic resistance to humans. Antimicrobial resistance (AMR) in the environment is influenced by heavy metals, biocides, antibiotic‐resistant genes, and antibiotics. Humans are exposed to antibiotic‐resistant bacteria and genes through a variety of pathways, including the consumption of animal and vegetable products and drinking water (Samreen et al. 2021).

Bacteria develop AMR through natural selection, adapting to evolutionary pressures over time (Baquero et al. 2021). The acquisition of AMR can be accelerated by selection pressure on bacteria caused by the unethical use of antibiotics for human health, aquaculture, livestock farming, agriculture, and antibiotic deposits in untreated hospital and pharmaceutical company effluents. Numerous studies have shown that specific categories of resistance‐inducing substances responsible for the antibiotic resistance evolution, such as antibiotics and nonantibiotic antimicrobial agents, such as metals and biocides (primarily surfactants and disinfectants), are well‐characterized and contribute to AMR (Figure 2) (Samreen et al. 2021).

The selection of resistant microorganisms caused by the use of biocides, herbicides, pesticides, and medications poses a major threat to the natural environment (S. S. Chung et al. 2018). Microorganisms may be subject to selective pressure from environmental AMR drivers, which could promote HGT and the spread of AMR. These drivers promote the proliferation of resistant microorganisms and their resistant genes through a variety of processes (Larsson and Flach 2022). MGEs like plasmids and transposons cocarry genes that provide resistance to chemicals, heavy metals, disinfectants, and antibiotics. Bacterial species can readily spread these MGEs (Martins and Rabinowitz 2020).

### 3.1 Antibiotic and Antibiotic Resistance Genes

The widespread and inappropriate use of antibiotics in medical, veterinary, and agricultural settings has had a major impact on AMR. Since the 1950s, antibiotics have been used extensively to cure diseases, and their use in fish farming and animal feed has grown dramatically as well (Kirchhelle 2018). Up to 50% of antibiotic prescriptions are thought to be unneeded or inappropriate. In many non‐European and non‐North American countries, antibiotics are still frequently taken without a prescription, accounting for 19% of total usage and sometimes almost 100% (Sohail et al. 2016).

Antibiotic contamination is a result of the insufficient waste treatment of nonmetabolized antibiotics and their residues, which raises antibiotic concentrations in the environment. Antibiotic contamination, a major contributor to AMR with far‐reaching global impacts, has emerged as a significant worldwide concern (Motlhalamme et al. 2024). The emergence of antibiotic resistance is mostly caused by the widespread use of antibiotics in healthcare settings. Hospital effluents serve as HGT hotspots, allowing ARGs to spread via MGEs, such as transposons and plasmids. These mechanisms allow ARGs to propagate between bacterial communities, further intensifying resistance. Overuse and abuse of antibiotics will eventually make it more likely that AMR will emerge, especially in bacteria that belong to the WHO priority pathogen category (Salam et al. 2023).

Antibiotics are frequently used in veterinary medicine as feed additives, growth promoters, and prophylactics in addition to treating infections. A substantial portion (30%–90%) of these antibiotics is excreted unchanged into manure, leading to environmental contamination (Samreen et al. 2021). These residues endanger human health by getting into the food chain through tainted animal products and contributing to the selection and dissemination of resistance genes within environmental microbiomes (Manyi‐Loh et al. 2018). Opportunistic pathogens like *E. coli* and *Klebsiella* spp. are commonly reported from hospital wastewater that is linked to human infections, while common resistant pathogens like *E. coli*, *Salmonella* spp., and *Campylobacter* spp. have been found in manure, soil, and water. The widespread usage of antibiotics accelerates AMR through selected pressures, which has serious repercussions for food safety and public health (Parvez and Khan 2018; Y. Xu et al. 2020).

### 3.2 Heavy Metals

Heavy metals act as significant contributors to AMR in the environment by applying selective pressures that encourage the persistence and dissemination of resistant bacteria. Metals like arsenic, zinc, copper, and cadmium are commonly found in agricultural, industrial, and clinical settings, where they are used as antimicrobials, growth enhancers, and in medical applications (Pal et al. 2017). These metals often coselect for ARGs through efflux pumps, reduced membrane permeability, and metal sequestration. The horizontal transfer of metal and ARGs throughout bacterial populations is made possible by the frequent presence of heavy‐metal resistance genes on MGEs, such as plasmids and transposons (Di Cesare et al. 2016).

**Figure 3:**
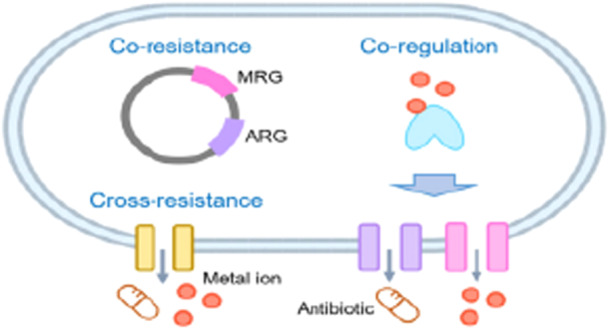
Mechanisms of antibiotic resistance caused by metals. Coresistance: MRGs and ARGs are physically associated within the same genetic element. Cross‐resistance: Antibiotic resistance gene (ARG) and metal resistance gene (MRG) are functionally related. Coregulation: When MRG and ARG are controlled by the same metal‐responsive regulator or signaling pathway (Z. Xu and Lin 2024).

The presence of heavy metals in wastewater, industrial discharges, and agricultural runoff increases selection pressures, promoting both cross‐resistance and coresistance (O'Malley et al. 2023). Cross‐resistance takes place when a single gene encodes a resistance mechanism, like efflux pumps, enabling protection against both antibiotics and metals. In contrast, coresistance occurs when multiple resistance genes, including those for metal and antibiotic resistance, are physically associated on MGEs, allowing their concurrent transfer (Gillieatt and Coleman 2024). Furthermore, coregulation mechanisms enable metals to serve as signals, triggering the expression of resistance genes for both antibiotics and metals (Figure 3) (Z. Xu and Lin 2024). These processes create AMR hotspots, highlighting the critical role of environmental pollutants in accelerating the global AMR crisis.

### 3.3 Biocides

**Figure 4:**
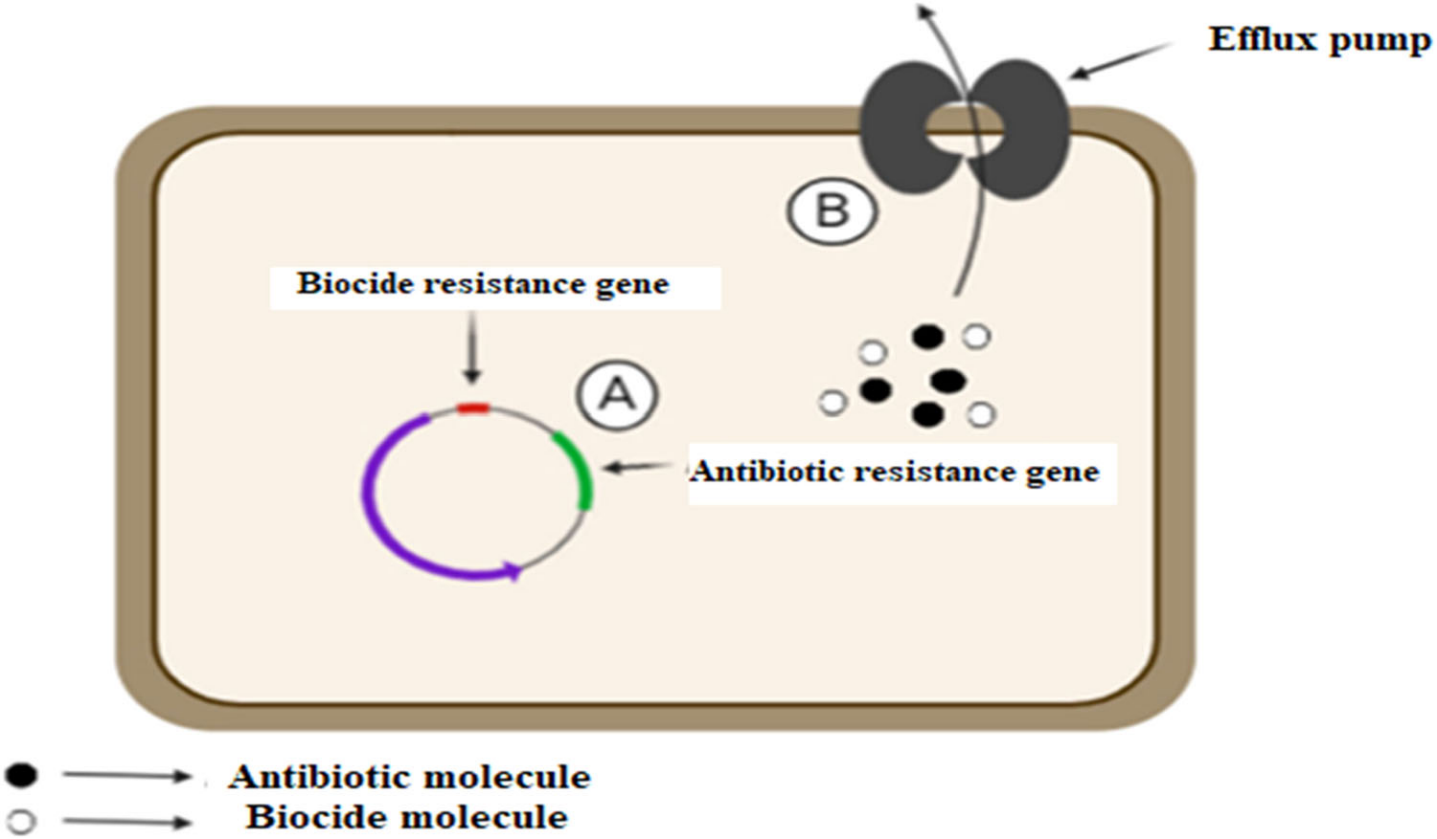
Coselection mechanisms in bacteria. (A) Coresistance occurs when genes conferring resistance to antibiotics and biocides are located on the same mobile genetic element, facilitating their concurrent transfer. (B) Cross‐resistance emerges when a single resistance mechanism, such as efflux pumps, provides defense against both antibiotics and biocides, allowing bacteria to survive exposure to multiple antimicrobial agents (Elekhnawy et al. 2020).

A class of compounds known as biocidal products has a broad spectrum of microbiostatic or microbicidal actions on various bacteria (Suganya et al. 2023). Biocides are commonly utilized in hospitals, household disinfectants, and veterinary settings (Kahrilas et al. 2015). Several routinely used biocides, such as triclosan, formaldehyde, ethanol, and chlorhexidine, are widely approved. Sublethal concentrations of biocides promote the selection of mutations that confer antibiotic resistance, similar to the selection of ARGs under sublethal antibiotic exposure. Bacteria may undergo stress responses in response to sublethal concentrations of biocides, which could result in mutations and the emergence of resistance (Feng et al. 2021). When biocides are misused, bacteria that are resistant to the available antibiotics may be selected (Feng et al. 2021). Biocide‐selected AMR involves mechanisms like cross‐resistance, where bacteria develop shared resistance traits such as efflux pumps or modified membrane permeability that defend against both biocides and antibiotics, and coresistance, where resistance genes for both are colocated on transferable genetic elements, facilitating their concurrent selection and spread (Figure 4) (Elekhnawy et al. 2020; Engin et al. 2023).

## 4 Hotspots for Antibiotic‐Resistant Bacteria

Among the ways that antibiotics can enter the environment are through the use of antimicrobial products, hospital effluents, human and animal wastes, and animal feed laced with antibiotics (Sivagami et al. 2020). Infected individuals who handle and process meat, such as farmworkers, can also spread the infection. In both pathogenic and nonpathogenic bacteria, these behaviors stimulate the creation of environmental hotspots that encourage the spread of antibiotic resistance. Agriculture and aquaculture operations, pharmaceutical manufacturing facilities, wastewater systems, and clinical settings like hospitals are important AMR hotspots (Sivagami et al. 2020). Hospital effluents in particular are significant reservoirs of resistant bacteria and resistance genes because of the high levels of antibiotic use in these settings. Therefore, hospitals and other clinical settings are seen as important AMR hotspots, where favorable conditions accelerate the development and spread of resistance (Shafiq et al. 2024).

A substantial amount of antibiotics given to food‐producing animals is excreted unmetabolized. Moreover, nonconsumed antibiotics present in animal feed can accumulate directly in soil sediments. Another major source of environmental antibiotic contamination is manure (Zalewska et al. 2021). Solid waste produced by animal farms is commonly used as farmyard manure or fertilizer for soil. However, a substantial amount of antibiotics from this manure leaches into aquatic systems, such as lakes and rivers, through surface runoff. Although antibiotic contamination from various sources is widely recognized, research on the fate and transport of antibiotics in animal excreta or stored manure remains limited. The accumulation of broad‐spectrum antibiotics in soil disrupts natural microbial communities, contributing to the emergence of resistant bacteria and ARGs. These changes present substantial risks to both human and environmental health (Rasheela et al. 2024).

Antibiotics are frequently employed as preventive measures, medicinal drugs, and feed additives in aquaculture. Seventy to eighty percent of these antibiotics are discharged into the environment. They build up in different environmental compartments as a result of inadequate digestion, which facilitates the development of antibiotic resistance in exposed bacterial populations by exerting selective pressure (Koirala et al. 2023). Antibiotics must be monitored for their metabolism and transformation, their entry into the food chain, and their residues in animal products and waste. One major factor contributing to the spread of AMR is the unchecked discharge of antibiotic residues into the environment. In addition to harming the ecosystems, this contamination interacts with more general environmental issues, including pollution, climate change, and biodiversity loss. All of these elements work together to reduce microbial diversity, which fosters the development and spread of AMR (Adegbeye et al. 2024).

## 5 Public Health Impact of Antimicrobial Resistance

AMR is a major global health concern that affects human health, reduces the effectiveness of medical treatments, and jeopardizes the long‐term sustainability of healthcare systems worldwide. As antimicrobial medication resistance rises in humans, livestock, and plants, minor infections can turn into life‐threatening conditions (Coque et al. 2023). The growing ineffectiveness of commonly used antibiotics against resistant bacteria poses a major threat to public health. AMR, which results in treatment delays, recurrent infections, and the dissemination of resistance across microbial populations, is one of the most urgent health concerns of the 21st century. According to current estimates, drug‐resistant infections claim the lives of 700,000 people annually. If AMR is not addressed, it is expected that 10 million deaths would occur yearly by 2050, with Asia and Africa bearing the brunt of the burden with an estimated 4.7 million and 4.2 million deaths, respectively (Dadgostar 2019; Kariuki 2024; Puri et al. 2025).

In Ethiopia, the abuse of antimicrobials is widespread among healthcare providers, untrained practitioners, livestock workers, and drug users. Studies have reported alarming levels of AMR, contributing to increased morbidity, mortality, and rising healthcare costs (MOH 2021). This growing resistance undermines the effective treatment of infections, leading to prolonged hospital stays, higher medical expenses, and significant public health challenges. If left unaddressed, the global economic burden of AMR could reach an estimated $100 trillion by 2050. Additionally, AMR poses a serious threat to achieving sustainable development goals, hindering progress in global health, economic stability, and overall well‐being (Chinemerem Nwobodo et al. 2022).

Infections that were previously treatable with standard antibiotics are becoming increasingly difficult to manage, resulting in prolonged illnesses and a higher risk of complications (Hay et al. 2018). Common infections, including bloodstream infections, pneumonia, and urinary tract infections, are showing growing resistance to first‐line antibiotics, leading to treatment failures, recurrent infections, and deteriorating health outcomes (Huemer et al. 2020). Moreover, AMR presents major challenges in conducting medical procedures and managing chronic diseases. Immunocompromised patients undergoing chemotherapy, organ transplantation procedures, or complex surgeries are particularly vulnerable to drug‐resistant infections, which can lead to poorer health outcomes and prolonged hospital stays (So and Walti 2022). Additionally, infections caused by drug‐resistant bacteria significantly increase healthcare expenses due to prolonged hospitalizations, the need for additional diagnostic procedures, and the reliance on costly treatment options. Addressing the rising global challenge of AMR requires the implementation of robust surveillance systems and preventive measures. Key strategies include early detection, prudent antibiotic usage, and stringent infection control practices, all of which are vital in curbing this growing public health threat (Founou et al. 2017).

## 6 Strategies to Combat Antimicrobial Resistance

The likelihood threat of an antimicrobial‐resistant era has prompted various policy measures, many of which emphasize the crucial role of personal decisions and behaviors in promoting the responsible use of antibiotics and preventing disease spread (Abimbola et al. 2021). Antibiotic resistance is often associated with the abuse of antimicrobial agents. To maintain the effectiveness of antibiotics, everyone in society needs to contribute collectively. Such an approach is the key to reducing antibiotic resistance. The rising challenge of AMR has made it essential to develop and implement effective strategies to tackle this issue (Gautam 2022). The worldwide scientifically accepted measures that are employed in the fight against AMR include the Global Action Plan, antibiotic stewardship initiatives, AMR monitoring systems, reliable diagnostic techniques, preventive infection control strategies, the concept of One Health, and innovative measures to antibiotics (Ferraz 2024).

### 6.1 Global Action Plan

In 2015, the WHO, in collaboration with the Food and Agriculture Organization of the United Nations (FAO) and the World Organization for Animal Health (OIE), developed the GAP to tackle AMR, following the One Health strategy (Mendelson and Matsoso 2015). The Global Action Plan promotes increased national awareness of AMR, targeting various groups within the human, animal, and agricultural sectors. Its objectives include implementing necessary measures to prevent the emergence and spread of resistant bacteria, reducing infection rates through improved sanitation, hygiene, and infection prevention practices; strengthening One Health surveillance; enhancing diagnostic methods for identifying and characterizing resistant bacteria; accelerating the development of new antibiotics, treatments, vaccines, and other interventions. As a result, numerous countries pledged to implement NAPs on AMR to tackle the issue of antimicrobial‐resistant infections following the GAP on AMR (Tang et al. 2023). Several nations, including Ethiopia, created their NAPs, aligning them with the AMR goals outlined in the GAP, to combat AMR through a One Health approach (Lota et al. 2022).

### 6.2 Antimicrobial Stewardship (AMS)

AMS is a multidisciplinary, multifaceted, and cooperative approach that involves healthcare leaders, microbiologists, clinical pharmacists, physicians, nurses, farmers, veterinarians, and infectious disease specialists to decrease the development of AMR, to progress patient treatment results and safety, and to make AMS attainable (Popoola 2024). AMS involves managing the use of antibiotics to preserve their effectiveness while ensuring they remain accessible to those in need. The establishment of AMS programs requires the creation of a dedicated and specialized team (Aslam et al. 2021).

The successful implementation of a robust AMS program, viewed through the One Health lens, must involve interventions aimed at influencing the prescribing practices of both human and animal healthcare professionals. Furthermore, there must be a concerted effort to encourage responsible antibiotic production practices, guarantee the proper disposal of unused antibiotics, and establish efficient waste management systems to ensure responsible antibiotic use across the entire product lifecycle. Additionally, the efficient use of antibiotics in veterinary medicine must be improved (Ashiru‐Oredope et al. 2022). Tackling AMR in animal health requires educating farmers and veterinarians on the responsible use of antibiotics, as well as fostering the development of alternative treatments for animal diseases (Doidge et al. 2020).

### 6.3 AS System

The AS system is a key strategic priority of the GAP to combat AMR (Do et al. 2023). According to the WHO, surveillance involves the ongoing, systematic collection, analysis, and interpretation of health‐related data, essential for the planning, implementation, and assessment of public health practices. AMR surveillance systems play a crucial role in tracking AMR across humans, animals, agriculture, and the environment. A well‐executed surveillance system helps determine the presence of AMR within a specific community. Effective AMR monitoring demands a reinforced multisectoral approach and streamlined coordination (Lim et al. 2021). Furthermore, thoroughly monitoring antimicrobial usage is strongly advised to minimize or prevent the emergence of ARB and their genetic elements. Microbial genomics and metagenomics play a vital role in addressing AMR across human, animal, and environmental sectors. They function as both surveillance systems and diagnostic tools, offering critical insights into the origins, transmission, and resistance mechanisms of pathogens (C. S. C. Liu and Pandey 2024). Therefore, the AMR surveillance system helps determine the extent of the AMR issue, detect emerging resistance, track the spread of specific resistance patterns, and link outbreaks to particular resistance mechanisms (Majumder et al. 2020).

### 6.4 Effective Diagnosis

Improving diagnostic capabilities is another crucial strategy in the battle against AMR. To identify and monitor AMR in humans, livestock, and the environment, reliable and effective diagnostic and public health laboratory methods are necessary. Rapid diagnostic testing is one example of an advanced diagnostic technology that can assist in reducing the needless use of antibiotics and support well‐informed medical decision‐making. These tests make it possible to gather patient data quickly, which helps determine the best course of treatment and reduces the need for unnecessary antibiotic prescriptions (Minejima and Wong‐Beringer 2016). Advanced genomic techniques like whole genome sequencing and polymerase chain reaction are laboratory procedures that help identify certain ARGs. This enables accurate infection therapy in a matter of hours as opposed to the days or weeks needed for conventional culture‐based techniques. The main use of genomics is the study of particular infections, resistance profiles, and gene alterations linked to resistance. Even in polymicrobial infections, numerous pathogens can be detected at the same time using metagenomics, which can also reveal previously undiagnosed resistance mechanisms (Avershina et al. 2021). Consequently, data produced in laboratories are crucial in advancing evidence‐based AMR therapies. Thus, laboratory‐generated data play a significant role in driving evidence‐based interventions in AMR (Jarocki et al. 2024).

### 6.5 Infection Prevention and Control (IPC) Practices

Preventing and managing diseases caused by resistant bacteria is essential in the fight against AMR (Haque 2020). IPC measures aim to minimize the spread of infections and resistant bacteria in healthcare facilities, public spaces, communal settings, and livestock farming. A primary focus of IPC in addressing AMR is infection prevention through the promotion of proper water, sanitation, and hygiene (WASH) practices (Haque 2020). Promoting WASH practices is crucial, particularly in hospitals, schools, universities, markets, and community settings. Maintaining proper hygiene, especially among healthcare workers, helps prevent the transfer of resistant bacteria, lowering the threat of antibiotic‐resistant infections in high‐risk groups. This practice can help prevent the transmission of resistant bacteria, particularly through the hands of healthcare workers, thereby decreasing the risk of ARB entering vulnerable patients. Experimental evidence supports that adhering to proper hand hygiene practices among healthcare workers can significantly reduce hospital‐acquired infections. Therefore, IPC aims to minimize infections and curb the transmission of resistant strains in communities, as well as in human and veterinary healthcare settings. Thus, following and maintaining proper hygiene measures is crucial in preventing the occurrence of infections (Mouajou et al. 2022).

### 6.6 One Health Strategy to AMR

The One Health strategy has developed into a holistic, multisectoral strategy that connects human, animal, and environmental health to tackle AMR. By encouraging collaboration across sectors, it addresses the complex and interconnected causes of AMR worldwide (Nardulli et al. 2023). To address AMR effectively, policies must involve coordination among healthcare, veterinary, and environmental policymakers. This collaboration should focus on initiatives like AMS programs in both human and veterinary medicine, environmental regulations to prevent antibiotic contamination in ecosystems, and the establishment of consistent regulatory frameworks across countries (Ferraz 2024). The improper use of antimicrobials in human healthcare, veterinary medicine, and agriculture plays a major role in the emergence and spread of MDR organisms in these sectors (Nardulli et al. 2023). Resistant bacteria can spread among humans, animals, and the environment, highlighting the need for a One Health strategy to combat AMR comprehensively. National Action Plans, supported by international organizations, such as the WHO, integrate the One Health strategy to reduce AMR, increase awareness, and encourage AMS (Ma et al. 2021).

In healthcare environments, antibiotics are often inappropriately prescribed for conditions like viral infections, which do not respond to such treatments. This misuse contributes to the development and spread of ARB within both community settings and healthcare facilities (Davis et al. 2017). To mitigate this problem, healthcare providers need to engage in thorough discussions with patients, offering clear explanations, accurate diagnoses, and considering alternative treatment options when appropriate (Lekshmi et al. 2017). In livestock farming, the widespread use of antibiotics for therapeutic purposes and as growth promoters has led to reservoirs of ARB, such as *E. coli*. These resistant strains can transfer to humans through direct contact or via the food chain. To address this issue, several countries have prohibited the use of antibiotics as growth promoters and are exploring alternatives, including bacteriophages, vaccines, and probiotics (Vora et al. 2023).

Environmental contamination is another significant contributor to AMR. Antibiotics and resistant microorganisms enter the environment through sewage discharge, farming runoff, and inappropriate pharmaceutical discharge. Once released into the environment, they persist and promote the selection of resistant microbes, creating reservoirs in water bodies, soil, and sediments. These reservoirs can transmit resistance back to the animals and humans (Collineau et al. 2023). To address environmental AMR, effective pharmaceutical waste management, reduced antibiotic usage, and enhanced waste treatment are essential. Hence, integrating AMR programs within the One Health framework is vital for tackling AMR across human, animal, and environmental health sectors (Martak et al. 2024). Surveillance systems that quantify AMU and AMR across these sectors provide critical data for integration and action. By fostering collaboration across disciplines, the One Health strategy offers a unified framework to combat AMR and safeguard global health (Bennani et al. 2021).

#### 6.6.1 Importance of Collaboration in One Health Concept

The One Health concept emphasizes how crucial it is to coordinate efforts across different sectors like healthcare, veterinary, agriculture, and environment to completely combat AMR. To address the underlying reasons for resistance and lessen its negative effects on public health, stakeholders can work together to implement measures (Shedeed 2024). The development of integrated monitoring systems that track antibiotic use and the transmission of resistance in the human, animal, and environmental domains is one of the most crucial components of the one health approach (Singh et al. 2024). However, a lot of current programs are not fully integrated and frequently function in a disjointed fashion. One major weakness in global efforts to combat AMR is the absence of frameworks that combine data from the fields of environmental, animal, and human health. To effectively tackle AMR, it is essential to implement collaborative policies, promote joint research to find innovative solutions, improve education and awareness, boost IPC measures, and share best practices, data, and resources across various sectors (Rhouma et al. 2023; Wernli et al. 2022).

### 6.7 Novel Alternative Therapies

Developing alternative therapeutic approaches to address the problem of AMR has become crucial due to the collapse of the antibiotic pipeline. New strategies are badly needed to prevent AMR and maintain the efficacy of antimicrobial agents. Numerous promising strategies are now being researched, including antimicrobial peptides (AMPs), nanotechnology, bacteriophage therapy, immunotherapies, probiotics, clustered regularly interspaced short palindromic repeats (CRISPRs)‐Cas system, and artificial intelligence (AI) (Olatunji et al. 2024; Rugarabamu and Mwanyika 2025). These innovative approaches offer restricted inhibition spectra for targeted therapy, which gives them an edge over antibiotics. They make it easier to choose medications that target particular bacteria, protecting useful bacteria and minimizing adverse microbiome effects (Walsh et al. 2021). Therefore, as AMR continues to escalate, developing innovative therapeutic and preventive strategies is essential to effectively tackle the challenges associated with its emergence.

#### 6.7.1 Antimicrobial Peptides

AMPs offer an encouraging approach in combating AMR due to their wide‐ranging potency and reduced likelihood of eliciting resistance. These naturally occurring compounds exert significant antibacterial, antiviral, and antifungal effects by damaging cell membranes (Lazzaro et al. 2020). Through a combination of mechanisms, AMPs can effectively destroy bacteria. They achieve this by disrupting cell walls and membranes, targeting intracellular functions, and acting against bacterial biofilms, leading to cell death. The mechanism involves some AMPs interfering with enzymes, like transglycosylases and transpeptidases, which are responsible for cross‐linking peptidoglycan strands. This prevents the formation of a rigid cell wall. The inhibition of peptidoglycan synthesis causes bacterial cells to become structurally weak, making them susceptible to osmotic lysis and finally resulting in cell death (Stojowska‐Swędrzyńska et al. 2023).

**Figure 5:**
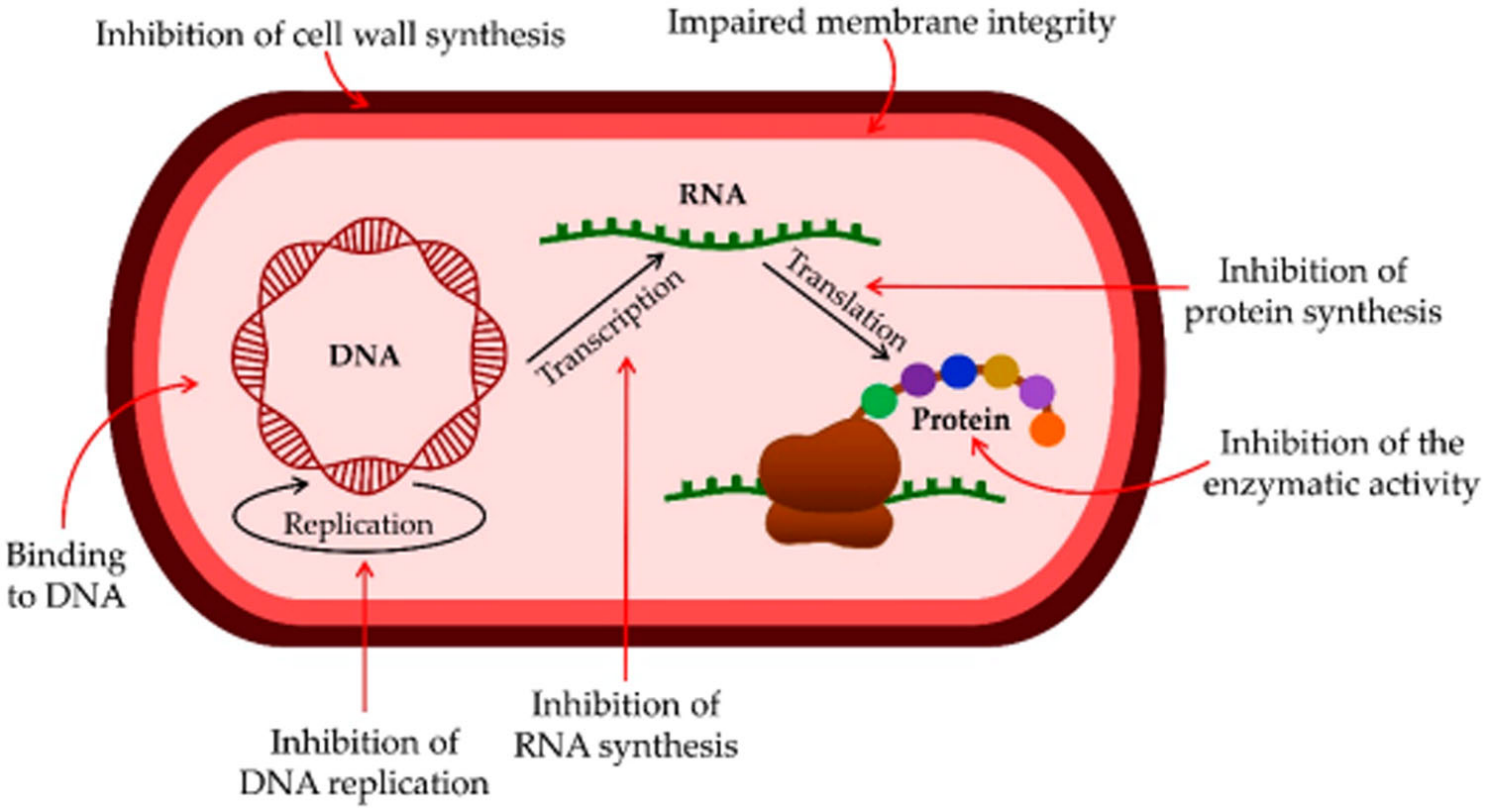
Antimicrobial peptides (AMPs) exert their bactericidal effects through multiple mechanisms. They disrupt the synthesis of the bacterial cell wall and impair cell membrane integrity, resulting in structural damage and increased permeability. Additionally, AMPs bind to nucleic acids (DNA and RNA) and ribosomes, inhibiting essential cellular mechanisms, such as DNA duplication, RNA formation, and protein production. These combined actions make AMPs effective against a wide range of bacterial pathogens. DNA, deoxyribonucleic acid; RNA, ribonucleic acid (Talapko et al. 2022).

Likewise, the interaction between positively charged AMPs and the negatively charged components of bacterial membranes results in pore formation, causing leakage of cellular contents and ultimately leading to cell death. Some AMPs target intracellular processes, particularly nucleic acids (DNA and RNA) and protein synthesis machinery. The AMPs bind to DNA, RNA, and ribosomes and then inhibit DNA, RNA, and protein synthesis, respectively (Figure 5) (Talapko et al. 2022). As potential alternatives to traditional antibiotics, AMPs hold significant promise in combating AMR. The Food and Drug Administration (FDA) of the United States has authorized several peptide‐based medications, highlighting their potential for therapeutic use. Nevertheless, further studies are necessary to gain a comprehensive understanding of AMPs' mechanisms of action and enhance their clinical applications (Nuti et al. 2017).

AMPs are promising alternatives to conventional antibiotics in combating AMR due to their broad‐spectrum activity. However, several challenges limit their clinical application (Saeed et al. 2022). The synthesis of AMPs is often expensive, making large‐scale production difficult. Additionally, many AMPs face stability issues in physiological conditions and may exhibit toxicity toward human cells. Furthermore, there is limited clinical data on their long‐term efficacy and safety, which hinders their widespread adoption in medical practice (Espinal et al. 2023).

#### 6.7.2 Nanoparticles (NPs)

**Figure 6:**
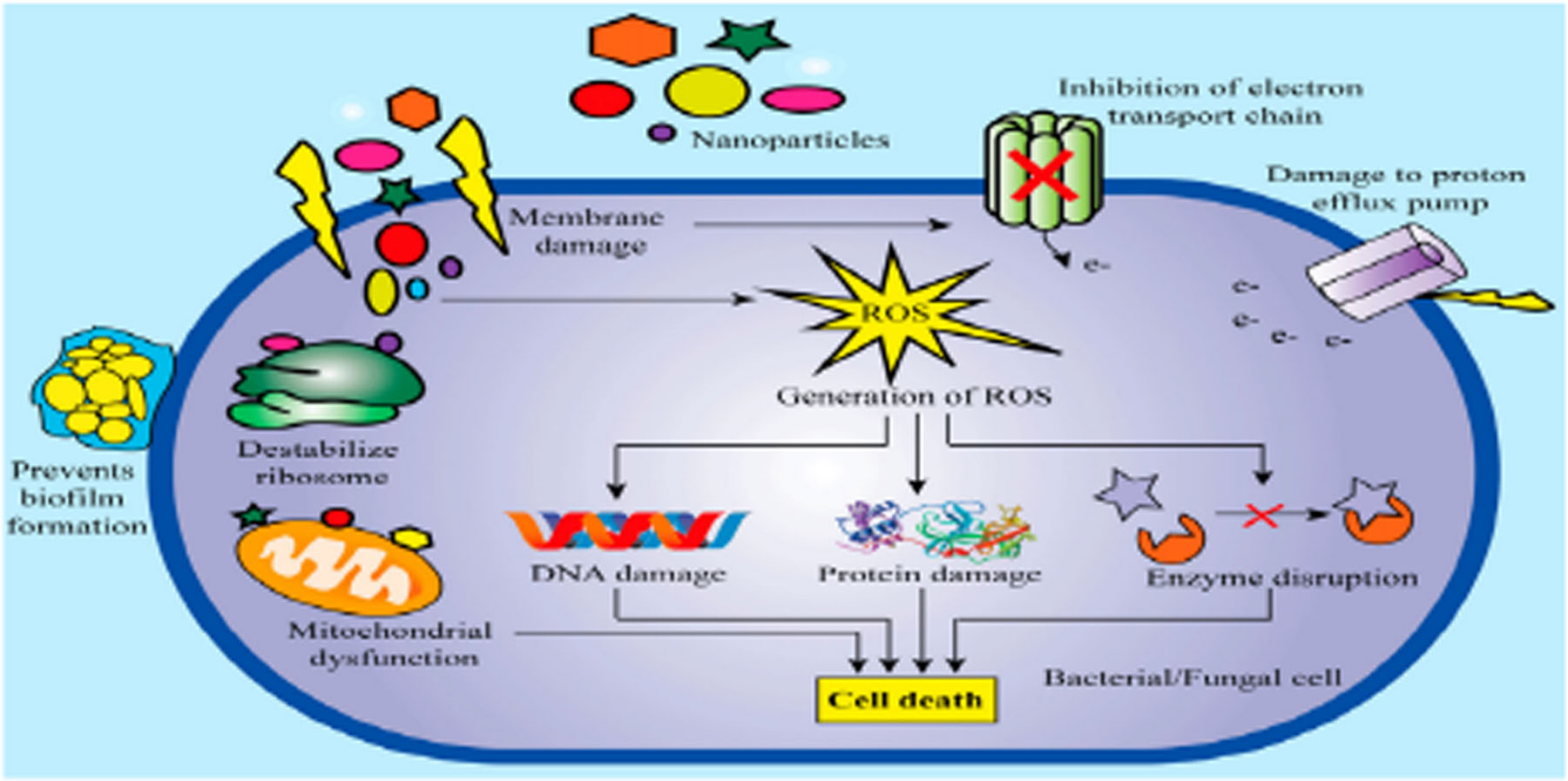
Nanoparticles (NPs) demonstrate antimicrobial effects against bacteria and fungi through various mechanisms. They directly affect the membranes or cell walls of bacteria and fungi, causing structural damage and preventing the formation of biofilms. Furthermore, NPs produce harmful reactive oxygen species (ROS), induce both innate and adaptive immunological responses, and cause intracellular disruptions such as enzyme inhibition, DNA damage, and protein malfunction to provide strong antibacterial and antifungal effects. Because of these complex processes, NPs show promise as agents to fight microbial infections (Sharmin et al. 2021).

NP‐based methods, which combine novel nanomaterial with classical antimicrobial therapies, have demonstrated promise in overcoming resistance in planktonic and biofilm phenotypes (Figure 6) (Sharmin et al. 2021). Antibiotic activity is increased by NPs, which also maintains the effectiveness of current medicines and provides new antibacterial action mechanisms (Lv et al. 2021). NPs can selectively target a single bacterial cell, increasing the effectiveness of antimicrobial agents and halting the development of resistance. Certain NPs, such as silver nanoparticles (AgNPs), zinc oxide nanoparticles (ZnONPs), and gold nanoparticles (AuNPs), are utilized to treat bacterial infections (Moo et al. 2020). Nanoparticles target resistant bacteria in a variety of ways, including disrupting membranes, generating reactive oxygen species (ROS), inhibiting biofilms, and interfering with genetic material. They are a promising response in the battle against AMR because of their multitarget strategy, which makes them extremely powerful against resistant bacteria and lowers the chance of resistance development (León‐Buitimea et al. 2022).

According to a recent study, antibacterial activity and the production of ROS by AgNPs are linked to cell wall and plasma membrane damage. These actions alter the structural integrity of the membrane, which disrupts the transport proteins and causes nutrient leakage (Hamouda et al. 2019). Another study reported that when AgNPs are combined with different antibiotics, such as gentamicin and chloramphenicol, they have a notable synergistic antibacterial effect for treating *Enterococcus faecalis* infections (Katva et al. 2018).

Nevertheless, nanotechnology‐based antimicrobials offer promising alternatives to conventional antibiotics to combat MDR pathogens; their application faces several challenges. Safety and environmental concerns remain significant, as NPs may accumulate in the environment and pose risks to human health (Ahmed 2025). Moreover, the high production costs limit their large‐scale use accessibility, and the uncertain long‐term effects of NP exposure raise questions about their sustainability and regulatory acceptance (Desai et al. 2025).

#### 6.7.3 Bacteriophages

Phage therapy makes use of bacteriophages, which are viruses that exclusively infect bacteria, to treat bacterial infections. Since they can cause bacterial lysis, lytic phages exhibit the most promise in therapy among the several types of bacteriophages (Garvey 2020). Phage treatment has become a feasible therapeutic approach, as the ability of antibiotics to combat AMR infections diminishes. Compared with traditional antibiotics, bacteriophages have several benefits, including high specificity, environmental safety, and potential uses beyond clinical settings. According to a study, phage therapy offers a promising line of defense against MDR bacterial infections brought on by *S. aureus*, *Pseudomonas aeruginosa* (*P. aeruginosa*), and *Acinetobacter baumannii* (Lin et al. 2017).

**Figure 7:**
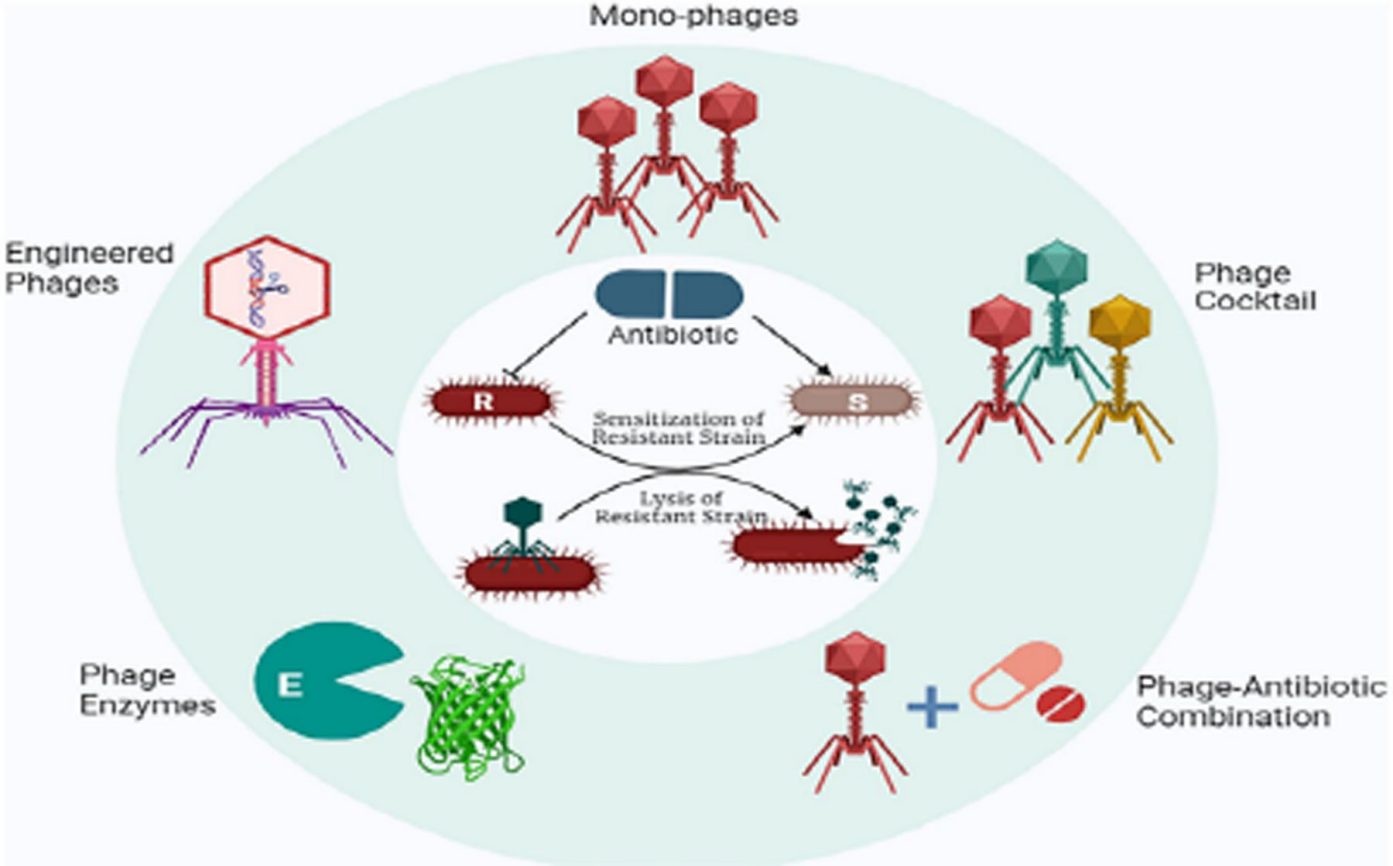
Phage therapy employs different strategies to combat resistant bacteria. Bacteriophages can either eliminate resistant bacterial strains through their lytic cycle or render them more susceptible to previously ineffective antibiotics. This dual mechanism enhances the promise of phage therapy in overcoming resistance to antimicrobials. R, resistant bacterial strain; S, sensitive bacterial strain (Pal et al. 2024).

Phages can precisely target and remove particular bacterial strains, even biofilm‐forming ones. One important method they have developed to break through and disturb biofilms is the synthesis of depolymerase (Di Martino 2018). Phage therapy can take several forms, such as monophage therapy, cocktail therapy, and the use of phage‐derived products like endolysins and depolymerases. Furthermore, combining phage therapy with traditional antibiotic treatments enhances its effectiveness. Overall, phages and their lytic proteins present significant potential for treating MDR bacterial infections, either as a sole treatment or in combination with antibiotics (Figure 7) (Pal et al. 2024).

Monophage therapy utilizes a single bacteriophage and shows significant promise for treating bacterial infections by specifically targeting individual bacterial strains, thereby effectively reducing pathogen populations without the broad‐spectrum effects associated with traditional antibiotics (Bozidis et al. 2024). However, the use of phage cocktails, combinations of many phages in a single formulation, received attention as a result of the quick emergence of bacterial resistance. This approach is advantageous because multiple phages can infect the same species or strain of bacteria, allowing for the targeting of various structural sites and metabolic activities. Consequently, phage cocktails are more effective than monophage therapy in minimizing the emergence of antimicrobial‐resistant mutant phages (Abedon et al. 2021; Karnwal et al. 2025).

The synergy between phages and conventional antibiotics has also been studied, with encouraging findings. Phage‐antibiotic synergy is the term for the phenomenon whereby subinhibitory concentrations of antibiotics increase phage productivity and decrease bacterial levels. This combined approach can even restore antibiotic sensitivity, as phages can bind to and degrade bacterial cell surface receptors responsible for antibiotic efflux, leading to resensitization of the bacterial cells (C. Liu et al. 2022; Pal et al. 2024). Phage therapy in combination with antibiotics has shown promise in treating infections brought on by MDR bacteria, including methicillin‐resistant *S. aureus* and pan‐drug‐resistant *K. pneumoniae* (Eskenazi et al. 2022). Apart from complete phages, phage‐derived products such as depolymerases, which break down bacterial capsules and biofilms, and endolysins, which break down bacterial cell walls, provide efficient substitutes for phages in the fight against bacterial diseases (Reuter and Kruger 2020). Furthermore, when natural phage hunting fails, genetically engineered phages offer enhanced antibacterial activity against MDR pathogens, providing a valuable alternative in the fight against resistant infections (Hussain et al. 2023).

Bacteriophage therapy is gaining renewed attention as a targeted approach to combat ARB, offering the advantages of high specificity against pathogenic strains (Subramanian 2024). However, unlike broad‐spectrum antibiotics that can act on a wide range of bacteria, phages often display a narrow host range, limiting their therapeutic scope. Furthermore, regularity uncertainty, lack of standardization, lack of standardized manufacturing processes, and the scarcity of large‐scale clinical trials create significant barriers to approval and widespread use (K. M. Chung et al. 2023). In contrast to antibiotics, which have well‐established production pipelines and regulatory frameworks, bacteriophage therapy still faces considerable scientific and clinical challenges before it can be fully integrated into routine medical practice (Hitchcock et al. 2023).

#### 6.7.4 Immunotherapy

In the battle against AMR, immunotherapy is a new strategy that shows promise as a substitute for conventional antimicrobials. The immune system is strengthened or altered via immunotherapy to combat infections (Umarje and Banerjee 2023). It may include the use of monoclonal antibodies (mAbs) and vaccines. mAbs are specially engineered proteins designed to target specific pathogens. Unlike antibiotics, which primarily disrupt critical bacterial functions, mAbs can bind to both essential and nonessential antigens, including virulence factors. This binding could lower the risk of resistance development, as mutations in these targets often lead to reduced virulence and enhanced clearance by the immune system (Mokhtary et al. 2022). In AMR, mAbs can neutralize bacteria or their toxins, providing a direct means of combating infection. Nevertheless, only a limited number of mAbs that fight bacteria have completed clinical studies. Three of these have been approved by the FDA thus far: bezlotoxumab, which prevents recurrent *Clostridium difficile* (*C. difficile*) infections, and raxibacumab and oblitoxaximab, which treat inhalational anthrax (Doroudian et al. 2024; Wilcox et al. 2017).

Vaccines are the most promising preventive measures to address the challenges of AMR because they can prevent infection by boosting the immune system's ability to identify and effectively respond to specific pathogens. They can also directly prevent infections caused by devastating AMR pathogens, and they have a huge potential impact on AMR by lowering the use of antibiotics and the selective pressure that leads to the emergence of resistant strains. Additionally, they reduce the use and reliance on antibiotics (Baker et al. 2018). Pneumococcal, *Neisseria meningitides* serogroups A, C, and ACYW, and *Hemophilus influenzae* type b vaccines are among the conjugate vaccines that are licensed worldwide. According to earlier research, bioconjugate vaccines are being developed to prevent a variety of pathogens that are resistant to antibiotics, including *Shigella*, *K. pneumoniae*, *S. aureus*, and *P. aeruginosa*. Some of these vaccine candidates have already advanced to the clinical phase (Sorieul et al. 2023; Talaat et al. 2021). However, immunotherapies have limitations, such as high development and treatment costs, rigorous safety testing requirements, and limited availability in low‐resource settings (Puri et al. 2025).

#### 6.7.5 Probiotics

Probiotics are helpful microbes that can prevent the growth of harmful bacteria and aid in restoring the gut flora. Probiotics exert their effects by generating short‐chain fatty acids from metabolic precursors, which results in similar downstream outcomes, including immune modulation and enhanced mucosal barrier function (Patel and DuPont 2015). In addition to physically occupying the epithelial niche and preventing other pathogens from colonizing the intestinal microbiota, probiotics may also have the added benefit of manufacturing their antimicrobial chemicals (Petrariu et al. 2024). According to recent studies, probiotics generate antimicrobial‐acting compounds called hydrogen peroxide and bacteriocins, which are inhibitory chemicals. Probiotics like *Bifidobacterium* and *Lactobacillus* species have been widely utilized to treat irritable bowel syndrome, traveler's diarrhea, and *C. difficile* infections. Many common infection‐causing pathogens, including *K. pneumoniae, P. aeruginosa*, *E. coli*, *S. aureus*, *Salmonella typhimurium*, and *Bacillus subtilis*, have been shown in various investigations to be either killed or rendered inactive by probiotics (Neidhöfer et al. 2023; Sniffen et al. 2018).

Although probiotics have shown promise in modulating the gut microbiota and decreasing the colonization of harmful bacteria, they have a limited impact on the fight against AMR. Limitations include strain‐specific efficacy, variations in colonization and survival within the gastrointestinal tract, and inconsistent clinical outcomes (Wani et al. 2025). Furthermore, some probiotic strains may carry transferable AMR genes, posing a potential risk of HGT. In addition, they lack large‐scale clinical data, regulatory monitoring, and standardization, which limit their wider use as a reliable strategy to combat AMR (Radovanovic et al. 2023).

#### 6.7.6 CRISPR‐Cas System

Untreatable bacterial infections claimed countless lives, underscoring AMR as a serious global health threat. This issue demonstrates the urgent need for action and highlights its critical public health significance. AMR is a life‐threatening phenomenon, driven by the misuse of antibiotics and the emergence of MDR superbugs. In response, international health organizations are calling for innovative solutions (H. Kadkhoda et al. 2024). One promising avenue is CRISPR‐Cas technology (CRISPRs‐associated proteins), a promising and versatile gene‐editing tool with wide‐ranging applications. CRISPR‐Cas systems can serve as antibacterial agents by restoring bacterial sensitivity to antibiotics through the precise targeting of resistance genes carried on the plasmids of pathogenic bacteria. Due to their accuracy and efficiency, these systems hold promise for controlling the spread of ARGs and eliminating bacterial virulence factors. This targeted gene elimination approach offers a valuable strategy for clinically managing ARG transmission and combating drug‐resistant infections (Saffari Natanzi et al. 2025). It offers opportunities for developing novel treatments, enhancing diagnostics, and creating new antimicrobial strategies, presenting an unprecedented chance to combat AMR effectively, with potential applications that extend beyond traditional antimicrobial strategies (Olatunji et al. 2024). According to studies, pathogenic bacteria with the CRISPR‐Cas system are less likely than those without these defense mechanisms to carry genes that cause antibiotic resistance (Hiva Kadkhoda et al. 2024).

Furthermore, combining CRISPR‐Cas technologies with existing therapeutic and preventive methods could strengthen efforts to manage and even reverse antibiotic resistance (Olatunji et al. 2024). Unlike traditional antibiotics, which are broad and often nonspecific, CRISPR‐Cas can be programmed to selectively target resistant bacteria, block resistance mechanisms, or inhibit biofilm formation (Saffari Natanzi et al. 2025). However, the application of CRISPR‐Cas technology is not without limitations. A key challenge lies in its dependence on specific protospacer adjacent motif sequences, which restricts the range of targetable sites. Off‐targets may also occur, causing unintended genetic changes. In addition, effective delivery of CRISPR components into diverse bacterial populations remains technically difficult, particularly in complex clinical environments. Ethical, safety, and regulatory considerations further complicate its transition from research to widespread clinical use (Alariqi et al. 2025; Kaupbayeva et al. 2024).

#### 6.7.7 AI in AMR Management

The health and welfare of the next generations depend on the effective management of drug resistance, which calls for a comprehensive approach that takes into account public health, economic, and scientific factors. The use of AI‐based machine‐learning methods and data‐driven technologies is essential in the fight against AMR (Alatawi et al. 2025; Yang et al. 2023). Advanced technologies facilitate predictive surveillance by processing vast clinical, genomic, and epidemiological data sets to identify emerging resistance patterns at an early stage. They also improve AMS by guiding clinicians toward evidence‐based drug choices, minimizing misuse, and overprescription. This strategy enhances patients' outcomes while minimizing the misuse of broad‐spectrum agents (Ali 2024). In addition, machine‐learning models can anticipate future resistance trends, enabling policymakers and healthcare providers to design protective measures for infection control and drug development. Overall, the integration of AI and data‐driven methods enhances the global fight against AMR by improving prediction, precision, and prevention (Gupta and Bhandary 2024; Pennisi et al. 2025).

While data‐driven tools and AI/machine learning offer promising solutions against AMR, they face notable challenges. Their effectiveness depends on access to large, standardized, and high‐quality data sets, which are often scarce or fragmented across regions, which hampers accurate model development (Sadana et al. 2025). Incomplete or biased data can lead to inaccurate predictions, while implementation depends on substantial infrastructure, expertise, and funding, which are barriers for many low‐resource settings. Moreover, the opaque nature of some machine‐learning models raises concerns about transparency, interpretability, and clinician trust. Regulatory hurdles, ethical considerations, and data privacy issues complicate their integration into routine clinical practices (Mennella et al. 2024; Rani et al. 2025).

## 7 Challenges to Combat Antimicrobial Resistance

Currently, tackling AMR faces several significant challenges. Key obstacles include a high disease burden, insufficient resources and funding, resistance to behavior change, limited laboratory capacity, inadequate surveillance systems, the emergence of natural resistance, challenges in data sharing, and weak leadership, governance, and coordination of AMS programs. These factors collectively hinder the successful implementation of AMR strategies (Mudenda et al. 2023).

In developing nations, in particular, high disease burdens raise the need for antibiotics to treat infections. Poor diagnostic facilities frequently result in the incorrect prescription of broad‐spectrum antimicrobials in high‐burden areas, even for diseases that are not bacterial (Dunachie et al. 2020). More human resources are needed, particularly in developing nations, which is one of the problems in implementing AMR plans into practice. Implementing successful AMR programs in these countries is hampered by a lack of funding and a shortage of medical experts with the necessary training to identify and manage AMR infections (Alhassan and Abdallah 2024). The successful use of effective AMR prevention measures is still hampered by these issues. Inadequate funds to implement policies like AMS campaigns into action are the other major obstacle to tackling AMR. In developing nations, since the majority of nations primarily rely on outside funds to establish and carry out AMR initiatives, this problem is especially noteworthy (Gandra et al. 2020).

Antimicrobial usage behavior of humans plays a crucial role in all aspects of AMR, including its prevention. The abuse of antimicrobials in both human and animal health, often driven by behavioral factors, significantly contributes to the rise of AMR. A dearth of behavioral change sustains self‐medication practices, which are major drivers of antimicrobial‐resistant infections (Othieno et al. 2020). Without addressing these behavioral issues, inappropriate prescribing, dispensing, and use of antimicrobials are likely to persist (Godman et al. 2021). Another major obstacle in combating AMR is the limited capacity of laboratories and ineffective surveillance systems. This issue is particularly pronounced in developing nations, where ensuring the delivery system and resource allocation for laboratory equipment, diagnostics, and reagents remains a significant hurdle (Vounba et al. 2022).

Significant challenges in assessing the worldwide burden of AMR include the scarcity of accurate microbial data, the gap between available data and clinical outcomes, and the unrepresentative nature of existing data (Mudenda et al. 2023). To estimate the spread of AMR, determine its causes, create focused control plans, and assess the effectiveness of strategies that have been put into place, accurate surveillance data is essential. Nonetheless, obtaining representative, high‐quality surveillance data is a challenge for many developing nations (Musa et al. 2023). Furthermore, because microbiological diagnostics are not widely used in clinical practice, there is a lack of trustworthy surveillance data in the setting of developing nations. In settings where culture and antibiotic sensitivity testing are not conducted, the problem of AMR becomes even more severe (Moirongo et al. 2022). Different studies reported that natural resistance poses a momentous challenge in controlling AMR, as it permits some bacterial species to inherently resist the effects of specific antibiotics. Natural resistance is an innate trait encoded in the genome of bacteria, unlike acquired resistance, which is the consequence of mutation or gene transfer (Nadeem et al. 2020). However, intrinsic resistance is enhanced due to exposure of bacteria to different antimicrobials. This characteristic restricts the efficacy of antibiotics against specific bacteria and makes treatment strategies more difficult (Moo et al. 2020).

Integrated information or data exchange is crucial for tracking, analyzing, and mitigating AMR (Fu et al. 2025). However, several challenges hinder seamless collaboration among various sectors and stakeholders, including healthcare, pharmaceutical industries, animal husbandry, agriculture, and environmental agencies. The use of diverse methods, formats, and parameters across sectors in AMR surveillance poses significant challenges for data integration and meaningful comparison. The absence of standardized data reporting frameworks leads to inconsistencies and gaps in the global AMR data set (WHO 2018). Many organizations and institutions also hesitate to share AMR‐related data due to privacy concerns, proprietary interests, and regulatory restrictions. The integrated health approach emphasizes collaboration between healthcare, animal farming, and environmental sectors. However, poor coordination among these sectors results in fragmented surveillance, reducing the effectiveness of AMR monitoring and intervention strategies (Singh et al. 2024). Thus, encouraging global collaboration and transparency among stakeholders and implementing an integrated AMR surveillance system will significantly improve efforts to combat AMR on a global scale.

The huge problem in addressing AMR is the scarcity of commitment among government leaders. For example, leaders at all levels must be involved in implementing NAPs on AMR, but this is not the case in most government units and healthcare facilities (Chua et al. 2023). Additionally, without this commitment, the fight against AMR becomes ineffective. Uncoordinated or inconsistent implementation of AMS activities impacts the fight against AMR (Alemkere et al. 2023). One of the hindrances to successful AMS programs in healthcare settings has been identified as ineffective interdisciplinary teams. The lack of functional AMS programs at certain healthcare facilities thus encourages the irrational prescription, dispensing, and administration of antibiotics (Baraka et al. 2019). Therefore, this makes it difficult to initiate and implement AMS activities.

## 8 Conclusions and Future Perspectives

The occurrence of drug‐resistant infections offers a key worldwide health constraint, affecting the health and well‐being of humans, animals, and the environment. This multifaceted issue is fueled by the excessive and incorrect use of antibiotics, metals, and biocides in healthcare, animal healthcare, fish farming, and agricultural activities, significantly accelerating the global spread of AMR. Medical facilities, pharmaceutical manufacturing sites, sewage treatment plants, and sites involved in the production of food and animals are major hotspots for bacteria and genes resistant to antibiotics. These hotspot sites are the main sources of resistant bacteria being released into the environment. Therefore, natural environments are essential for promoting genetic material exchange across bacterial species, which speeds up the spread of antibiotic‐resistant determinants between pathogenic and nonpathogenic microorganisms. This widespread transmission increases public health risks by producing infections that are difficult to control and associated with high rates of morbidity and mortality.

There is a significant information gap about how antibiotic resistance affects the health of humans, animals, and the environment, which emphasizes the need for more research. It has been thought that comprehensive global measures are necessary to address the growing threat of AMR. Enhancing National Action Plan following the Global Action Plan, encouraging responsible antibiotic use, establishing strong surveillance systems, implementing IPC measures, offering precise diagnostics, and developing new therapeutic agents are some of the plans. The concept of one health, which promotes cooperation between the environment, animal, and human health sectors, has been acknowledged as being essential to combat AMR. For the implementation of these integrated strategies, a multidisciplinary effort is thought to be essential, guaranteeing global collaboration and the development of innovative solutions. Antimicrobial efficacy can be maintained by implementing these strategies, protecting public health, and ensuring environmental sustainability for future generations.

## Author Contributions

**Kindu Alem:** conceptualization, writing original draft. **Mulat Dagnew, Mucheye Gizachew, Baye Gelaw, and Feleke Moges:** review and editing. All authors have read and approved the final version of the manuscript.

## Ethics Statement

The authors have nothing to report.

## Conflicts of Interest

The authors declare no conflicts of interest.
